# Analysis of HIV-1 diversity, primary drug resistance and transmission networks in Croatia

**DOI:** 10.1038/s41598-019-53520-8

**Published:** 2019-11-21

**Authors:** Maja Oroz, Josip Begovac, Ana Planinić, Filip Rokić, Maja M. Lunar, Tomaž Mark Zorec, Robert Beluzić, Petra Korać, Oliver Vugrek, Mario Poljak, Snježana Židovec Lepej

**Affiliations:** 1 University of Zagreb School of Medicine, Zagreb, 10000 Croatia; 2 University Hospital for Infectious Diseases “Dr. Fran Mihaljević”, Zagreb, 10000 Croatia; 3 Ruđer Bošković Institute, Zagreb, 10000 Croatia; 4 Division of Molecular Biology, Department of Biology, Faculty of Science, University of Zagreb, Zagreb, 10000 Croatia; 5 Institute of Microbiology and Immunology, Faculty of Medicine, University of Ljubljana, Ljubljana, 1000 Slovenia

**Keywords:** Epidemiology, Molecular medicine

## Abstract

Molecular epidemiology of HIV-1 infection in treatment-naive HIV-1 infected persons from Croatia was investigated. We included 403 persons, representing 92.4% of all HIV-positive individuals entering clinical care in Croatia in 2014–2017. Overall prevalence of transmitted drug resistance (TDR) was estimated at 16.4%. Resistance to nucleoside reverse transcriptase inhibitors (NRTIs), non-nucleoside RTI (NNRTIs) and protease inhibitors (PIs) was found in 11.4%, 6.7% and 2.5% of persons, respectively. Triple-class resistance was determined in 2.2% of individuals. In addition, a single case (1.0%) of resistance to integrase strand-transfer inhibitors (InSTIs) was found. Deep sequencing was performed on 48 randomly selected samples and detected additional TDR mutations in 6 cases. Phylogenetic inference showed that 347/403 sequences (86.1%) were part of transmission clusters and identified forward transmission of resistance in Croatia, even that of triple-class resistance. The largest TDR cluster of 53 persons with T215S was estimated to originate in the year 1992. Our data show a continuing need for pre-treatment HIV resistance testing in Croatia. Even though a low prevalence of resistance to InSTI was observed, surveillance of TDR to InSTI should be continued.

## Introduction

Croatia is a South-Eastern European country with a low prevalence of HIV-1 infection (<0.04%). A total of 1540 persons have been diagnosed with HIV-1 infection in the period 1985–2017^1^. Clinical care for HIV-infected persons is centralized, meaning that all persons diagnosed with HIV/AIDS in the country are followed at the University Hospital for Infectious Diseases (UHID) in Zagreb, Croatian capital^2^. All clinical, socio-demographic, virological and molecular data from patient’s medical records are collected at one place, enabling us to perform extensive countrywide epidemiological, virological as well as clinical studies of HIV infection.

HIV-1 is a retrovirus characterized by a high mutation rate and extensive genetic variability. HIV-1 strains are classified into four groups (M, N, O, P) and group M is responsible for the global pandemic^3,4^. Group M is divided in 9 genetically distinct subtypes (A-D, F-H, J and K), sub-subtypes (A1, A2, F1, F2) and 98 circulating recombinant forms (CRFs)^5^. Global distribution of HIV-1 subtypes is highly heterogeneous, but the most prevalent subtype in Central and Western Europe is HIV-1 subtype B, responsible for 85% of new HIV-1 cases^6,7^. Infection with HIV-1 subtype B in the population of men who have sex with men (MSM) is the most common factor that contributes to the spread of HIV-1 infection in Central and Western Europe, while the infection with non-B subtypes is usually related with heterosexual contact and immigration^8–10^. In Croatia, first cases of HIV-1 infection were linked to labour migrants who returned from Western European countries (infections with subtype B) and seafarers who acquired HIV in Africa and Eastern Asia (infections with non-B subtypes and CRFs)^2^. However, transmission routes in Croatia have changed considerably in the last two decades, in a way that 40–50% of new HIV-1 cases were attributed to MSM transmission during the time period 1985–2010^11–13^, while the latest national reports revealed that 83% of newly diagnosed HIV-1 cases in 2016 and 92% of new cases in 2017 acquired infection through MSM contact^1^.

Despite being a country with a low level of HIV epidemic, a previous national study showed a very high prevalence of transmitted drug resistance (TDR) of 22.0% (study period 2006–2008) in Croatia^14^, while the European SPREAD study, which included data for 25 European countries and Israel, showed an overall prevalence of 8.3% (study period 2008–2010)^15^. In contrast to Croatian data, regional countries reported substantially lower TDR prevalence, such as Slovenia (2.4%), Serbia (8.8%), Bulgaria (5.2%) and Greece (6.0%), while in Romania (14.8%), Hungary (17%) and northern Italy (12%) TDR prevalence is comparable to the Croatian setting^16–22^.

In the previous national study primary resistance to reverse transcriptase inhibitors (RTIs) and protease inhibitors (PIs) was analysed^14^. The most frequently found mutation was the revertant T215S (14.4%), associated with resistance to nucleoside analogues reverse transcriptase inhibitors (NRTIs), while TDR associated with resistance to non-nucleoside reverse transcriptase inhibitors (NNRTIs) was detected in three persons (2.4%) and primary resistance to protease inhibitors (PIs) was not reported during that study period^14^. The majority of persons were infected with HIV-1 subtype B (89%) and phylogenetic inference identified eight local transmission clusters (TC), among which the largest TC harboured T215S mutation and consisted of 19 Croatian patients^14^.

Due to the previously reported high TDR prevalence in Croatia and the fact that there is a predominant epidemic occurring among MSM at the local level, in this study we characterized HIV transmission and drug resistance dynamics, geographic distribution and evolutionary origin of TC in Croatia by the use of phylogenetic and phylodynamic analysis^23–30^. We included >90% of treatment-naive patients who entered clinical care during a 4-year period, 2014–2017. We analysed HIV-1 diversity, prevalence of TDR with the use of Sanger sequencing and deep sequencing and identified HIV-1 transmission networks using Maximum Likelihood phylogenetic and Bayesian evolutionary analysis.

## Results

### HIV-1 infection in croatia is driven by young MSM infected with subtype B

In total 436 HIV-infected persons entered clinical care at the Croatian Reference Centre for HIV/AIDS between January 2014 and December 2017. Here we report results for 403 (92.4%) persons, including 381 men (94.5%), 21 women (5.2%) and one transgender person (0.3%). The year of HIV diagnosis was concordant with the year of entry into care in 377 (93.5%) persons. There were 81 persons diagnosed at the recent stage of HIV infection and 322 persons diagnosed at the chronic stage of HIV infection. Persons entered clinical care at a median age of 36.0 years, with a median plasma viraemia of 131,951 copies/ml and a median CD4^+^ T cell count of 325.0 cells/μL (Table 1). The main risk factor for HIV infection was MSM (n = 358, 88.8%), followed by heterosexual contact (n = 39, 9.7%) and injecting drug users (IDU) (n = 3, 0.7%). The predominant HIV-1 subtype in this cohort was subtype B, found in 91.3% (368/403). Subtype A1 was determined in 4.2% (17/403) and subtype C in 1.7% (7/403), followed by recombinants CRF02_AG in 1.2% (5/403), CRF01_AE in 0.5% (2/403) and CRF06_CPX in 0.2% (1/403) (Table 1). HIV-1 subtype could not be assigned to three sequences, REGA Subtyping Tool defined them as recombinants of A1-B (2/403, 0.5%) and A1-C (1/403, 0.2%).

**Table 1:** Baseline characteristics of treatment-naive HIV-1 persons in Croatia who entered clinical care in the period 2014–2017.

Persons, n	403
**Gender, n (%)**	
Male	381 (94.5)
Female	21 (5.2)
Transgender	1 (0.3)
**Transmission risk, n (%)**	
MSM	358 (88.8)
Heterosexual	39 (9.7)
IDU	3 (0.7)
Unknown	3 (0.7)
**Reported country of infection, n (%)**	
Croatia	348 (86.4)
Outside Croatia	52 (12.9)
Unknown	3 (0.7)
**Stage at HIV diagnosis, n (%)**	
Recent infection	81 (20.1)
Chronic infection	322 (79.9)
**AIDS defining illness, n (%)**	
Yes	75 (18.6)
No	328 (81.4)
**Year of HIV diagnosis, n (%)**	
<2014	12 (3.0)
2014	91 (22.6)
2015	106 (26.3)
2016	100 (24.8)
2017	94 (23.3)
**Year of entry in clinical care, n (%)**	
2014	96 (23.8)
2015	106 (26.3)
2016	101 (25.1)
2017	100 (24.8)
**With SDRMs, n (%)**	
Total	66 (16.4)
2014	20 (20.8)
2015	16 (15.1)
2016	14 (13.9)
2017	16 (16.0)
**HIV subtype, n (%)**	
A1	17 (4.2)
B	368 (91.3)
C	7 (1.7)
A1-B	2 (0.5)
A1-C	1 (0.3)
CRF02_AG	5 (1.2)
CRF01_AE	2 (0.5)
CRF06_cpx	1 (0.3)
**Age at HIV diagnosis, n (%)**	
18–29 years	112 (27.8)
30–39 years	156 (38.7)
40–49 years	86 (21.3)
≥50 years	49 (12.2)
**Residence, n (%)**	
Zagreb	200 (49.6)
Outside Zagreb	203 (50.4)
Age, median (Q1-Q3) years	36 (29.0–43.0)
**Baseline CD4**^**+**^ **T cells/μL, n (%)**	
<100	88 (21.8)
101–250	71 (17.6)
251–400	90 (22.3)
401–600	82 (20.3)
>601	72 (17.9)
Baseline CD4^+^ T cells/μL, median (Q1-Q3)	325.0 (142.0–515.5)
Log_10_ baseline plasma viraemia, median (Q1-Q3)	4.9 (4.4–5.5)

MSM: men who have sex with men; IDU: injecting drug users; Q1, Q3: first and third quartile; n: number of individuals.

### High prevalence of resistant HIV-1 strains in Croatian cohort

The overall prevalence of surveillance drug resistance mutations (SDRMs) was estimated at 16.4% (n = 66/403) (Supplementary Table S1). Specifically, we identified 46/403 (11.4%) individuals with SDRMs to NRTIs. The most frequent NRTI mutations were: T215S (7.4%), L210W (2.7%), T215D (1.7%) and M41L (1.2%). Resistance to NNRTIs was determined in 27/403 (6.7%) cases, with mutations K101E (3.7%), K103N (2.5%) and L100I (1.9%) most frequently observed. Resistance to PIs was detected in 10/403 (2.5%) persons, most often due to mutations V32I (1.9%) and I47V (1.9%). Nine persons (2.2%) were found with triple-class SDRM pattern (NRTI + NNRTI + PI). For extensive review of all identified SDRMs see Supplementary Table S1. Resistance to integrase strand-transfer inhibitors (InSTIs) was detected in single person, G140A, 1% (1/100). Furthermore, analysis of the integrase region revealed 3 accessory resistance mutations (T97A, Q146QH, D232N). According to the Stanford Genotypic Resistance Interpretation Algorithm, v8.8^31^ all TDR mutations that we identified were clinically relevant except mutation T69D, while the International AIDS Society (IAS) list confirmed nearly all mutations, except T215S, K219R, G190E and G140A, as clinically relevant^32^. The majority of persons with TDR were MSM infected with subtype B (64/66, 96.9%), two persons were infected with subtype B through heterosexual contact and two persons with TDR were infected with subtypes A1 and C, respectively (Supplementary Table S1). Persons infected with resistant HIV-1 strains, as compared to persons without primary resistance, were significantly more likely to be MSM (P = 0.042) and were less likely to be diagnosed with AIDS-defining illness (P = 0.002) (Table 2).

**Table 2:** . Comparison of clinical, socio-demographic and virological characteristics between: (1) All persons; (2) Transmission clusters; (3) Out of transmission clusters; (4) Without SDRM; (5) With SDRM.

	All persons	Persons in TC	Persons outside TC	P-value	Persons without SDRM	Persons with SDRM	P-value
Persons, n (%)	403 (100)	347 (86.1)	56 (13.9)	0.192	337 (83.6)	66 (16.4)	0.223
Gender, n (%)^b^							
Male	381 (94.5)	330 (95.1)	51 (91.1)		316 (93.8)	65 (98.5)	
Female	21 (5.2)	16 (4.6)	5 (8.9)		20 (5.9)	1 (1.5)	
Transgender*	1 (0.3)	1 (0.3)	/		1 (0.3)	/	
Risk factor, n (%)^a^				<0.0001			0.042
MSM	358 (88.8)	319 (91.9)	39 (69.6)		294 (87.3)	64 (97.0)	
Heterosexual	39 (9.7)	24 (6.9)	15 (26.8)		37 (10.9)	2 (3.0)	
IDU*	3 (0.7)	2 (0.6)	1 (1.8)		3 (0.9)	/	
Unknown*	3 (0.7)	2 (0.6)	1 (1.8)		3 (0.9)	/	
Reported country of infection n (%)^a^				0.014			0.495
Croatia	348 (86.4)	305 (87.9)	43 (76.8)		294 (87.2)	59 (89.4)	
Abroad	52 (12.9)	39 (11.2)	13 (23.2)		41 (12.2)	6 (9.1)	
Unknown	3 (0.7)	3 (0.9)	/		2 (0.6)	1 (1.5)	
Stage at HIV diagnosis, n (%)^a^				0.009			0.670
Recent infection	81 (20.1)	77 (22.2)	4 (7.1)		69 (20.5)	12 (18.2)	
Chronic infection	322 (79.9)	270 (77.8)	52 (92.9)		268 (79.5)	54 (81.8)	
AIDS defining illness, n (%)^a^				0.039			0.002
Yes	75 (18.6)	59 (17.0)	16 (28.6)		61 (18.1)	2 (3.0)	
No	328 (81.4)	288 (83.0)	40 (71.4)		276 (81.9)	64 (97.0)	
HIV subtype, n (%)^a^				<0.0001			0.066
B	368 (91.3)	329 (94.8)	38 (67.9)		303 (89.9)	64 (97.0)	
non-B	35 (8.7)	18 (5.2)	18 (32.1)		34 (10.1)	2 (3.0)	
Primary resistance, n (%)^a^	66 (100)	61 (17.6)	5 (8.9)	0.140	/	66 (100)	
Age, median (Q1-Q3), years^c^	36 (29.0–43.0)	35.0 (28.0–43.0)	39.0 (33.0–45.0)	0.019	36.0 (29.0–43.0)	37.0 (28.0–45.8)	0.681
Baseline CD4^+^ T cells/μL^c^, median (Q1-Q3),	325.0 (142.0–515.5)	325.0 (148.3–532.5)	305.0 (95.0–460.0)	0.155	330.0 (148.0–515.0)	275.5 (89.8–533.5)	0.253
Log_10_ baseline plasma viraemia^c^, median (Q1-Q3)	4.9 (4.4–5.5)	4.9 (4.4–5.5)	4.9 (4.2–5.4)	0.633	4.9 (4.4–5.5)	5.01 (4.4–5.7)	0.156

TC: transmission clusters; SDRM: surveillance drug resistance mutation; MSM: men who have sex with men; IDU: injecting drug users; Q1, Q3: first and third quartile; n: number of individuals; a: associations for categorical variables were tested using Chi-squared test; b: associations for categorical variables were tested using Fisher’s exact test; c: associations for continuous variables were tested using Mann-Whitney Wilcoxon test; *variables with small number of samples were excluded from the sum of proportions and the statistical analysis.

### Comparison of SDRM detected with Sanger sequencing (SS) and deep sequencing (DS)

We compared patterns of SDRMs detected by Sanger sequencing (variant frequency threshold of approximately <15%) and deep sequencing (variant frequency threshold >5%). A total of 48 persons were included in this part of the study, but sequencing of one sample failed. SS and DS did not detect any SDRM in 31 samples (65.9%). We identified SDRMs in 12 samples by SS (25.5%) and in 16 samples by DS (34%) (Supplementary Table S2). As we defined complete mutation concordance when both SS and DS identified the same pattern of SDRMs, in 50% (8/16) of samples results completely matched. Partial concordance was identified in 2 samples, both of them carried triple-class drug resistance with the same pattern that was identified by SS. DS analysis detected all of these mutations and additionally identified several other RTI mutations that were present at frequencies below the SS threshold. Complete divergence was observed in 6 samples. In 4/6 samples complete divergence was determined in samples in which DS detected T215S mutation at frequencies below the SS threshold (around 10%). In the other two samples divergence was assigned due to the different patterns of SDRMs detected by SS and DS (Supplementary Table S2).

### The character of Croatian HIV-1 transmission networks is mainly local and regional

Phylogenetic trees were constructed separately for 368 subtype B sequences, 17 subtype A1 sequences and 7 subtype C sequences. For sequences subtyped as intersubtype recombinants (n = 3) and CRFs (n = 8) phylogenetic analyses were not performed, due to the low number of samples In total, 54 TCs were determined by phylogenetic analysis; namely, 45 (83.3%) of subtype B, 7 (13%) of subtype A1 and 2 (3.7%) of subtype C. Individuals observed in TC, as compared to individuals outside TC, were more likely diagnosed at recent stage of infection (odds radio (OR), 3.7; 95% confidence interval (CI), 1.3–14.5; P = 0.009) and younger age (P = 0.019), infected with HIV-1 subtype B (OR, 9.2; 95% CI, 4.4–19.3; P = <0.0001), acquired HIV-1 infection due to the MSM transmission (OR, 5.1; 95% CI, 2.3–11.1; P = <0.0001) and reported Croatia as a probable country of infection (OR, 2.4; 95% CI, 1.1–4.9; P = 0.014) (Table 2).

**Figure 1:**
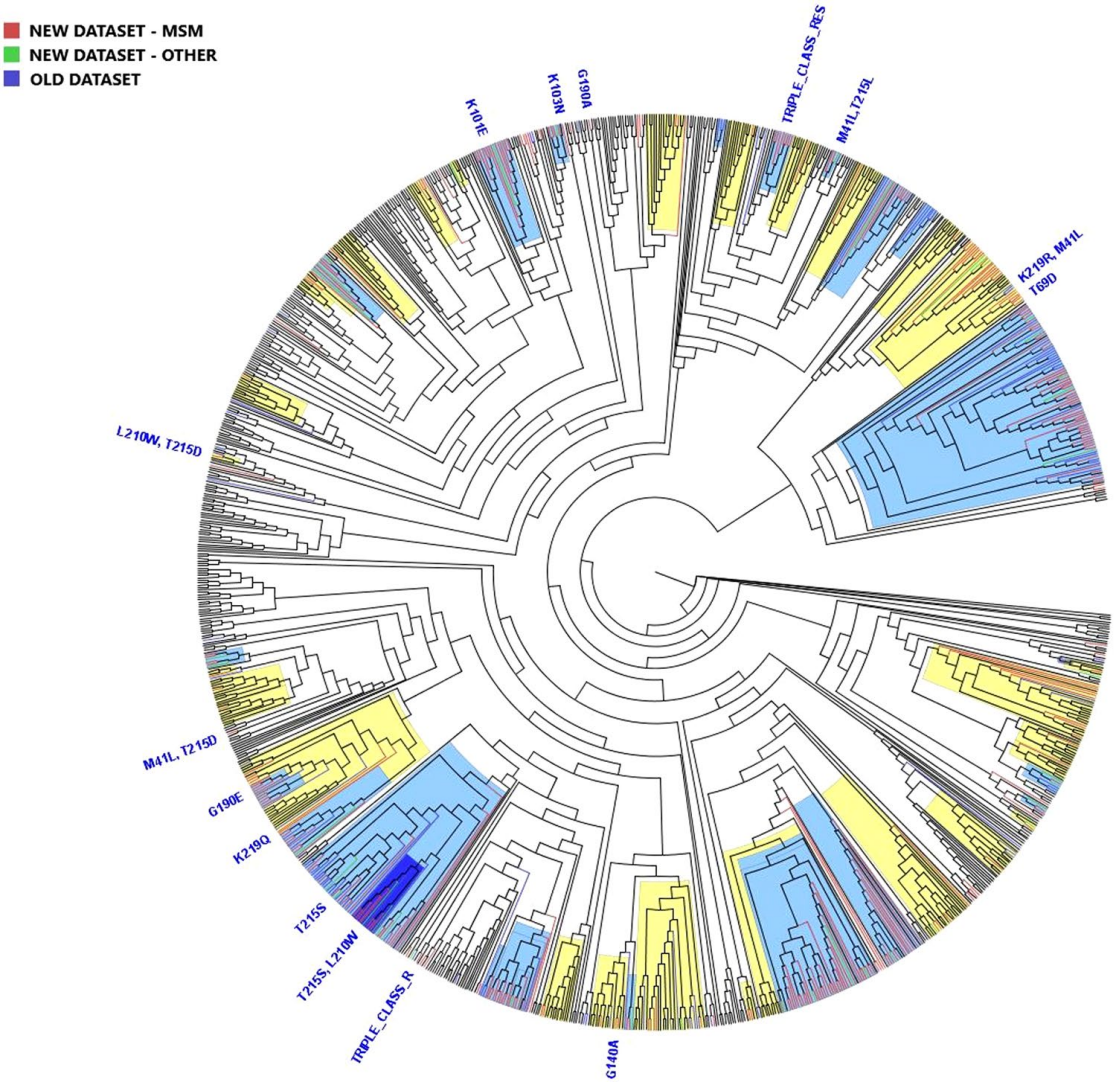
Maximum likelihood phylogenetic analysis of the Croatian HIV-1 subtype B sequences from the old (2006–2008), the new (2014–2017) datasets and background sequences. Branches of Croatian sequences from the new dataset are coloured according to transmission risk: red, men who have sex with men (MSM); green, other. Branches of sequences from the old dataset are coloured blue, while branches of all background sequences are coloured black. All identified surveillance drug resistance mutations (SDRMs) are positioned on the tree along with the corresponding sequences. For SDRMs T215S; T215S + L210W; K101E; M41L + T215L; V32I + I47V + T215D/E + K103N + L100I (TRIPLE_CLASS_RES) that form local transmission clusters (TCs), above each cluster corresponding SDRMs were noted, meaning that all sequences inside TCs harbour SDRMs. TCs with >75% of Croatian sequences (local clusters) are highlighted blue, while TCs with <75% of Croatian sequences (mixed clusters) are highlighted yellow.

The subtype A1 phylogenetic tree revealed that 41.1% (n = 7) of sequences belonged to one local TC (containing 4 Croatian sequences) and one mixed TC with non-Croatian sequences (containing 3 Croatian sequences), with approximate likelihood ratio test value (aLRT) >0.90. In addition, 5 mixed TCs each containing only one Croatian sequence were identified, indicating a separate introduction of these strains in the country (Supplementary Fig. S1). Inference analysis of subtype C determined presence of two local transmission pairs with aLRT >0.90 and two mixed TCs, each containing only one Croatian sequence (Supplementary Fig. S2). Phylogenetic analysis of subtype B sequences included >90% of the analysed cohort and consisted of 368 local sequences from the new dataset (2014–2017), 107 local sequences from the previous dataset (2006–2008) and 663 background sequences (Fig. 1). Sequences from the previous time period (2006–2008) were included in order to identify “active” TCs, consisting of sequences from the previous (2006–2008) and new (2014–2017) datasets, “historical” TCs, consisting of sequences from the previous dataset and “newly formed” TCs, consisting of sequences from the new dataset. Overall, the analysis revealed that 89.4% (329/368) of sequences from the new dataset were a part of 23 local TCs and 22 mixed TCs with non-Croatian sequences. We identified 9 (20%) TCs with 3 persons, 2 (4.4%) TCs with 4 persons, 23 (51.1%) TCs with 5 to 15 persons and 11 (24.4%) TCs that include ≥16 persons. The size of the TCs ranged from 3 to 80 persons. We selected 15 TCs with ≥5 Croatian sequences and presented their characteristics in Table 3. Most of the identified TCs were active (43/45), since analysis revealed only two historical TCs. Out of 43 active TCs, there were 15 clusters expanding throughout the observed time period and 28 were designated as newly formed TCs. Among the newly formed TCs, 10 were local and 18 also included non-Croatian sequences.

**Table 3:** Characteristics of 15 selected transmission clusters comprising of ≥5 Croatian sequences subtype B with aLRT >0.90 and their estimated times of the most recent common ancestor.

Cluster, total no. of sequences, (n)	Croatian sequences, n (%)	aLRT	Geographic origin of non-Croatian sequnces in TC	SDRMs in TC, (n)	Type of cluster	Full dataset analysis			Individual cluster analysis		
						tMRCA, mean	tMRCA, median	tMRCA, 95% HPD	tMRCA, mean	tMRCA, median	tMRCA, 95% HPD
1 (80)	77 (96)	0.99	Poland, Slovenia	/	Expanding	1998.3	1998.5	1994.1–2002.2	1998.5	1998.6	1994.7–2001.8
2 (53)	52 (98)	0.97	UK	T215S (38), T215S + L210W (9)	Expanding	1992.1	1992.3	1988.7–1997.1	1993.01	1992.9	1988.4–1997.9
3 (51)	40 (76)	0.91	Poland, Slovenia, Czech Republic, US	/	Newly formed	2003.3	2003.4	2006.6–2011.8	2003.8	2003.9	2006.6–2011.8
4 (46)	32 (70)	0.95	Germany	K219Q (1), G190E (1)	Expanding	1990.4	1990.9	1985.4–1995.1	1995.5	1995.6	1993.8–1996.9
5 (33)	28 (85)	0.94	Slovenia, Germany, Peru	K219R + M41L (1); T69D (1)	Expanding	1993.2	1993.3	1987.2–1999.7	1993.01	1992.9	1988.4–1997.9
6 (21)	20 (95)	0.9	Slovenia	/	Newly formed	2005.3	2005.5	2001.9–2008.3	2004.7	2004.8	2001–9–2007.5
7 (17)	13 (76)	0.96	Serbia	/	Newly formed	2005.1	2005.4	2001.7–2008.1	2004.5	2004.7	2001.4–2007.1
8 (14)	14 (100)	0.94	/	K101E (14)	Newly formed	2008.7	2008.8	2005.8–2011.6	2009.1	2009.2	2006.7–2011.3
9 (14)	12 (86)	0.97	Slovenia, UK		Expanding	1996.2	1996.4	2002.7–1989.6	1998.3	1998.6	1992.8–2003.1
10 (9)	8 (89)	0.93	UK	V32I + I47V + T215D + K103N + L100I (8)	Newly formed	2008.2	2008.1	2005.9–2010.6	2006.9	2007.1	2004.1–2009.7
11 (30)	14 (47)	0.99	Slovenia	/	Expanding	1992.3	1992.4	1986.7–1995.5	ND	ND	ND
12 (11)	11 (100)	0.96	/	T215S (2), T215S + L210W (9)	Expanding	2009.1	2009.2	2006.6–2011.5	ND	ND	ND
13 (8)	8 (100)	0.99	/	/	Newly formed	2007.2	2007.4	2002.3–2011.6	ND	ND	ND
14 (24)	10 (42)	0.98	Czech Republic, Slovakia, Germany	/	Expanding	1992.5	1992.2	1987.5–1998.1	ND	ND	ND
15 (21)	5 (24)	0.99	Slovenia	/	Newly formed	1995.7	1995.9	1992.1–1999.6	ND	ND	ND

No: number; aLRT: approximate likelihood ratio test value; TC: transmission clusters; SDRM: surveillance drug resistance mutation; tMRCA: times to the most recent common ancestor; HPD: highest posterior density; ND: not done.

All subtype B sequences carrying SDRMs (n = 64) were included in the phylogenetic analysis. Forward transmission of SDRMs throughout the period 2014–2017 was determined in several TCs: (1) T215S (n = 20) and its sub-cluster T215S + L210W (n = 9) (2), K101E (n = 14) and (3) V32I + I47V + T215D/E + L100I + K103N (n = 8). A comparison of clinical, demographic and molecular characteristics of these TCs consisting sequences of only the new dataset (2014–2017) is given in Table 4. The most common SDRM determined in the previous dataset (2006–2008), T215S, remained the most common SDRM in the newly studied period; specifically, 53 sequences from the previous and the new datasets formed one TC. Within this TC, a sub-cluster with SDRMs T215S + L210W expanded during the time frame of the new dataset (2014–2017). We identified one local TC with a triple-class resistance pattern. This TC was formed of eight Croatian sequences and one UK sequence. The Croatian sequences contained the SDRM pattern PI: V32I, I47V + NRTI: T215E/D + NNRTI: L100I, K103N. The sequence originating from the UK had a similar mutation pattern with additional SDRMs (PI: M46L, V82A and NRTI: T215Y). Also, one Croatian patient with a complex pattern of resistance (SDRM to PI: I84V + NRTI: M184MIV, T215S, L210W + NNRTI: K101E, Y181C, G190A, P225PH) was observed in a transmission pair with a Serbian sequence with a similar SDRM pattern with one additional SDRM (PI: M46I), indicating cross border transmission of multi-class drug resistance. For some of the sequences with SDRMs (n = 9) we did not find forward transmission. Some of these sequences carrying SDRMs: T69D, G190E, K219Q and K219R + M41L belonged to large TCs (Table 3), others were not part of TCs, such as SDRMs K103N, G140A, G190A and M41L + T215D, while the sequence with SDRMs T215D + L210W formed a transmission pair with a Croatian sequence from the previous dataset (2006–2008) that carried the same resistance pattern.

**Table 4:** Main characteristics of Croatian transmission clusters harbouring SDRM.

	T215S cluster	T215S + L210W cluster	K101E cluster	V32I + I47V + T215D/E + K103N + L100I cluster
Total number of individuals, n (%)	20 (100)	9 (100)	14(100)	8 (100)
Estimated time of the TC origin, years	1992	2009	2008	2008
**Gender, n (%)**				
Male	20 (100)	9 (100)	12 (85.7)	8 (100)
Female	/	/	1 (5.9)	/
Transgender	/	/	1 (5.9)	/
**Transmission risk, n (%)**				
MSM	20 (100)	9 (100)	13 (92.9)	8 (100)
Heterosexual	/	/	1 (7.1)	/
**Reported country of infection, n (%)**				
Croatia	18 (90)	9 (100)	12 (85.7)	7 (87.5)
Abroad	2 (10)	/	1 (5.9)	1 (12.5)
Unknown	/	/	1 (5.9)	/
**Stage at HIV diagnosis, n (%)**				
Recent infection	4 (20)	/	1 (7.1)	2 (25)
Chronic infection	16 (80)	9 (100)	13 (92.9)	6 (75)
**AIDS defining illness, n (%)**				
Yes	6 (30)	1 (11.1)	4 (28.6)	/
No	14 (70)	8 (89.9)	10 (71.4)	8 (100)
Age, median (Q1-Q3), years	37.0 (29.0–40.0)	26.0 (21.8–26.5)	47.5 (43.0–52.5)	29.5 (25.3–35.5)

SDRM: surveillance drug resistance mutation; n: number of individuals; TC: transmission cluster; MSM: men who have sex with men; Q1, Q3: first and third quartile.

**Figure 2:**
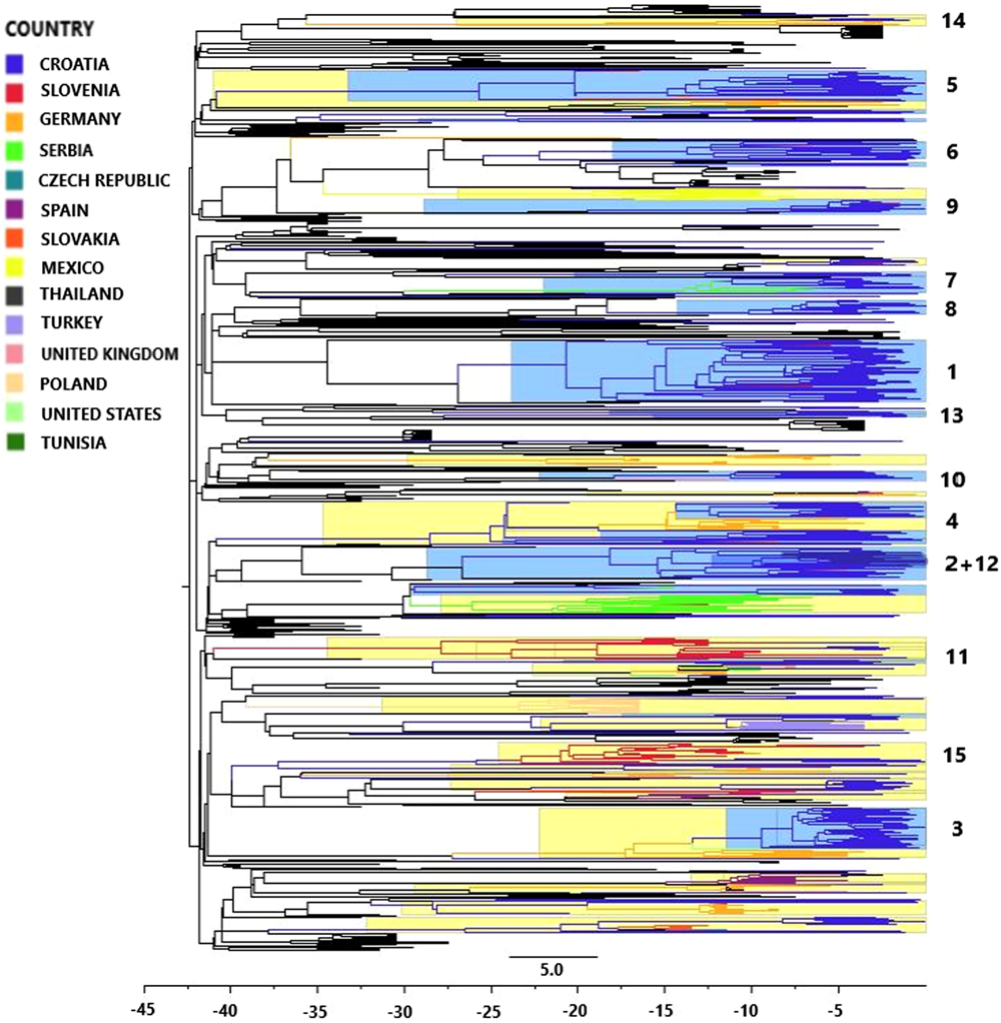
Bayesian maximum clade-credibility tree of the Croatian HIV-1 subtype B sequences from the new dataset (2014–2017) and corresponding background sequences. Branches are coloured according to the geographic origin, as presented on the legend of Fig. 2, with the exception of less frequent background sequences, which are coloured black. TCs with >75% of Croatian sequences (local clusters) are highlighted blue, while TCs with <75% of Croatian sequences (mixed clusters) are highlighted yellow. TCs with ≥5 Croatian sequences (Table 3) are marked on the sideward by ordinal numbers. The scale is set at 5-year intervals starting at the sampling time of the latest sequence (2017.91).

We inspected the geographical origin of non-Croatian sequences found in TCs, as well as the sampling dates, and observed that most of the TCs with non-Croatian sequences are very diverse (Fig. 2 and Supplementary Fig. S3). Non-Croatian sequences originated mainly from Germany, Slovenia, Serbia and Czech Republic. In addition, some Croatian sequences clustered with sequences from the United States (1 TC, n = 17 sequences), Turkey and the United Kingdom (1 TC, n = 12 sequences), Spain and Tunisia (1 TC, n = 11 sequences) and Mexico (1 TC, n = 11 sequences).

Phylogenetic inference was repeated with 43 SDRM sites present in sequences and showed that all transmission TCs remained stable with the exception of one local TC, comprised of 3 sequences from the new dataset and 3 sequences from the previous dataset. When SDRM sites were present in the phylogenetic inference, this TC merged with 5 sequences originating from Poland and formed a mixed TC.

### First croatian HIV-1 transmission clusters originate from early 90 s

Bayesian skyline analysis was performed in order to determine when the HIV-1 epidemic started in Croatia by estimating the time to the most recent common ancestor (tMRCA) of the HIV-1 sequences belonging to the Croatian TCs. This analysis was carried out only for subtype B sequences from the new dataset (n = 356) and their corresponding control sequences (n = 571). The phylogenetic tree, constructed using Bayesian skyline analysis (Fig. 2), was in good agreement with the phylogenetic tree obtained by Maximum Likelihood analysis (Fig. 1). Importantly, none of the TCs identified initially, using Maximum likelihood phylogenetic analysis, broke down during the Bayesian skyline analysis. Estimated tMRCA values for the 15 TCs with ≥5 Croatian sequences are presented in Table 3.

Our results suggested that the earliest persistent introduction of HIV-1 to Croatia could have occurred in the year 1990 and heralded the expansion of Cluster 4 (95% highest posterior density (HPD), 1985.4–1995.1). Significant transmission of variants carrying SDRMs probably occurred later on, by the progenitors of TCs 2, 8, 10 and 12 (Table 3). The progenitor of the largest SDRM TC, Cluster 2, dated to the year 1992 (95% HPD, 1988.7–1997.1). Cluster 12, with the SDRM T215S + L210W, appearing as a sub-cluster within Cluster 2, emerged at a more recent time, with its progenitor dated to the year 2009 (95% HPD, 2006.6–2011.5). The root node of Cluster 8, carrying the SDRM K101E, dated to the year 2008 (95% HPD, 2005.8–2011.6) and Cluster 10, responsible for the transmission of triple class drug resistance in Croatia, appeared to have originated in the year 2008 (95% HPD, 2005.9–2010.6).

## Discussion

In this study we characterized the Croatian HIV-1 epidemic by combining clinical, socio-demographic, molecular and virological data from treatment-naive HIV-1 persons. We also described HIV transmission networks responsible for the forward spread of HIV-1 infection and drug-resistant variants in the country. This study included >90% of HIV-1 persons entering HIV clinical care in the time period 2014–2017, therefore it is to the best of our knowledge the most extensive HIV molecular epidemiology study performed in Croatia, so far. Our results confirmed previous findings that in recent years HIV-1 infection in Croatia primarily affects the young MSM. Individuals were more likely to present for clinical care at the chronic stage of HIV-1 infection with a median CD4 count of 325 cells/μL. According to the international definition of late presentation for care^33^, which is defined by all persons with CD4 count <350 cells/μL, our cohort consisted of >50% late presenters and 18% were diagnosed with clinical AIDS. Even though this proportion is alarming, when compared to the study results from the period 1999–2004^34^, with baseline median CD4 count of 276 cells/μL and 34% persons presenting with clinical AIDS, our data show that the situation has improved.

The Croatian HlV-1 epidemic is distinguished by a high prevalence of TDR, as first reported in our previous nation-wide study (22%)^14^ and confirmed in the present study (16.4%). Even though TDR prevalence appears to have decreased moderately in the last decade, the pattern of SDRMs has changed significantly and become more challenging for tailoring a successful first-line treatment. In the previous study, high TDR was mainly due to one large TC of MSM with SDRM T215S, while NNRTI resistance-associated mutations were only present at a low frequency (2.4%) and PI resistance-associated mutations were not detected at all. In contrast, the present study shows that the revertant T215S remains the most frequent SDRM, determined in 30 (7.4%) persons. With this, TDR to NRTIs was the most prevalent, determined in 46 (11.4%) persons; however, we also observed the emergence of SDRMs to NNRTIs among 27 (6.7%) persons. In addition, PI resistance was determined in 10 (2.5%) persons, including 9 (2.2%) with triple-class drug resistance, mediated by the mutation pattern PI: V32I, I47V + NRTI: T215D/E + NNRTI: L100I, K103N. Phylogenetic inference indicated a transmission link with one UK sequence with a similar mutation pattern, PI: V32I, M46L, I47A, V82A + NRTI: T215Y + NNRTI: L100I, K103N. Moreover, a patient with treatment failure due to this resistance pattern has not yet been encountered in clinical care in Croatia, indicating that this SDRM pattern was introduced in Croatia from abroad and has subsequently spread in the country.

Prevalence of TDR in European countries has been monitored via the SPREAD program, organised under the supervision of the European Society for Translational Antiviral Research (ESAR)^15^. The latest SPREAD study analysed data for the period 2008–2010 and showed an overall TDR prevalence of 8.3%. The prevalence of SDRMs to NRTIs was 4.5%, while SDRMs to NNRTIs and PIs were present at lower frequencies, 2.9% and 2%, respectively. Comparing our data (Croatian old and new dataset) to the SPREAD data, we observed that overall TDR prevalence in Croatia was and remains high above the European average. TDR prevalence studies in the region show that only a few countries report high TDR prevalence similar to that of Croatia. However, one has to take into account that variation in the study design, national coverage and interpretation algorithms may affect cross-country comparison. Andreis *et al*. reported TDR of 12.3% in a cohort of 750 HIV-infected persons diagnosed in northern Italy in the time period 2013–2016, with E138A mutation being the most frequent^22^. However, Andreis *et al*. used the interpretation algorithm for genotypic drug resistance incorporated in Viroseq HIV Genotyping System, which also includes polymorphic mutations that do not indicate TDR. This highlights the importance of using a universally adopted mutation list for TDR surveillance, such as the WHO list^35^. In respect to that, E138A was also found in our cohort, but was not reported in this study. Temereanca *et al*. showed a 14.75% TDR prevalence in Romania^20^. The study included 61 newly diagnosed patients that enrolled in clinical care in the time period 1997–2011. Mezei *et al*. analysed TDR prevalence in Hungary (2008–2010) in a cohort of 30 persons and estimated the prevalence at 16.6%^21^. Even though both countries reported high TDR, study bias is possible due to the low number of samples included in their analyses.

The European SPREAD study analysed the presence of InSTI-resistant variants in Europe (2006–2007) before the introduction of InSTIs^36^. The study showed that before the drugs were introduced there were no circulating variants that could jeopardize the effect of InSTIs. InSTIs are drugs with very high potency that ensure fast and sustainable drop of viral load but also provide tolerability and safety for long-term use. Because of their positive characteristics they are recommended as a first-line regimen by ART guidelines^37^. Since the use of these drugs became more widespread recently, the emergence of InSTI-resistant variants can be expected^38^. However, surveillance of InSTI resistance is currently recommended only for patients with evidence of TDR to other drug classes^38^. Due to the high prevalence of TDR to RTI and PI drug classes in our cohort, we tested resistance to InSTIs in selected patients. InSTIs were first introduced to Croatia in 2015 and there was no clear benefit of baseline TDR testing to InSTI in persons who entered clinical care in the period 2014–2016. In addition, first cases of resistance in InSTI-treated persons were reported in 2016^39^, therefore InSTI resistance was tested only among treatment-naive persons who entered clinical care during 2017. We found only one major InSTI mutation (G140A), three accessory resistance mutations (T97A, Q146QH, D232N) and a very high prevalence of polymorphism S230N (49/100, 49%), which is not associated with reduced InSTI susceptibility. We confirmed high susceptibility to InSTIs in this Croatian cohort, as expected and reported by other countries^22,40–42^.

Subtype B remains the most common HIV-1 subtype in Croatia. The previous analysis^14^ determined infection with the subtype B in 89% of newly diagnosed HIV-1 persons and the present study showed a comparable prevalence rate of 91.3%. Among the non-B subtypes only subtype A1, subtype C and CRF02_AG variants were observed in >1% of the cohort. In respect to that, we focused more on transmission networks of subtype B. In the 2006–2008 analysis, phylogenetic inference identified eight distinct TCs and the largest TC consisted of MSM carrying T215S mutation^14^. Since no background sequences were included, only local TCs were identified and HIV introduction events remained unknown. The present study, which included sequences from the old (2006–2008) and new (2014–2017) datasets as well as control sequences, identified 45 TCs. The majority of TCs were active and only two clusters were comprised of only the sequences from the old dataset. Even though a considerable number of active TCs (65%) were newly formed, the clusters identified in the previous study expanded significantly and represented the largest TCs determined in this analysis. Most of the old dataset sequences that were determined to be without an obvious transmission link in the previous analysis, were observed to belong to mixed TCs, consisting of mainly regional (Slovenian, German, Czech, Poland and Serbian) and Croatian sequences in the present analysis. Most of these mixed TCs were intermixed with sequences from regional countries, however, it seems that a number of persons could have acquired the infection in other countries, such as Spain, Turkey, Mexico and/or the United States. Interestingly, we observed no connection between Croatian and Hungarian sequences and very few between Croatian and Italian sequences. Similar observations of cross-border HIV transmission have been described by other studies, together with reporting that risk factors, such as the use of alcohol/drugs prior to sex, visiting sex parties and travelling abroad to engage in sex, seem to be increasing in Europe^16,43,44^.

Overall, the phylogenetic analysis has shown that a significant number of persons (86%) were part of TCs, among which 70% were found in local TCs. When combining results of phylogenetic inference with socio-demographic data reported by persons included in this study, our results indicate that HIV-1 epidemic in Croatia is mainly local and driven by young MSM. Similar findings were observed in Slovenia, where Lunar *et al*. determined a high percentage of clustering among Slovenian sequences (81%)^30^. Our findings are comparable with epidemiological studies from other Western and Central European countries, where HIV-1 infection is concentrated among MSM infected with subtype B, however the proportion of clustered sequences was <60%^25–28,45,46^. Since analysis of TCs is dependent on sampling methods, methodological approaches and transmission cluster definition^47^, high extent of clustering in our study can be explained by high sampling density. This study included >90% of HIV-1 persons who entered HIV clinical care during consecutive 4-year period, while the majority of above mentioned studies achieved a coverage of 30–60%, sampled during an extended time frame (usually during a 10–20 year period).

A considerable number of persons carrying SDRMs (61/66, 92.4%) were observed in TCs, thus confirming the substantial forward spread of SDRMs in the country. Most of the sequences with SDRMs (83.6%) formed local TCs, of which the T215S cluster of 53 persons remained the largest SDRM cluster in Croatia. Phylodynamic analysis suggested that this TC could be one of the earliest clusters in the country, with its estimated tMRCA in the year 1992. T215 revertants are one of the most common NRTI mutations found in untreated persons^15,48^. These variants develop from strains containing T215Y/F mutations, primarily selected under the stavudine (d4T) and zidovudine (AZT) treatment and tend to persist for many years^49^. In Croatia, regimens containing AZT and d4T were in use from the early 90 s on, but the use of AZT has decreased to <5% in 2017 and d4T is not in use since 2012. However, the high prevalence of the revertant T215S variants in the Croatian HIV-1 cohort is likely a hallmark of once highly prevalent therapy in Croatia.

Along with T215S, we report on K101E, T215S + L210W and triple-class drug resistance pattern, as major contributors to the spread of resistant strains in Croatia. All strains carrying these mutations were found in local TCs. Interestingly, each cluster had its specific characteristics such as age of the persons participating in the cluster, prevalence of AIDS at initial diagnosis and estimated time of the origin of the cluster. However, one common characteristic was observed in all TCs – all persons but one were MSM. The TC with K101E, the triple-class resistance TC and the sub-cluster T215S + L210W were all identified as newly formed clusters. On the other hand, the largest TC with T215S is an expanding cluster that has contributed in the spread of HIV-1 infection in Croatia over the last two decades. Its longevity and impact on the local epidemic shows the importance of monitoring TDR on a national level. Particularly alarming is the finding of a local TC with triple-class resistance pattern. We estimated that the origin of this TC dates in 2008, with the sequence most closely related originating from the UK, sampled in 2006. Since it is a newly formed TC comprised of 8 Croatian sequences that were sampled throughout the period 2014–2017, we can expect to see more patients with this complex resistance pattern in Croatia in the future. For some of the observed SDRMs further transmission was not detected. These were possibly individually introduced into the country or originated from Croatian persons with treatment failure and simply had not been transmitted further, due to lower fitness of these variants. For mutations M46I, T69D, G190E, K219Q and K219R reduced fitness compared to wild-type virus has been observed, as these tend to revert back to wild type^50^. All persons with these SDRMs participated in mixed TCs, but none of the other related sequences, sampled in Croatia or abroad, shared these SDRMs.

In addition to Sanger sequencing, 48 randomly selected samples were tested for SDRMs using deep sequencing. The aim was to observe the agreement between the two sequencing platforms and estimate the potential use of DS for routine diagnostics of clinical HIV resistance. The proportion of resistant variants present in initial infection can fall below the standard level of detection (<15%) of SS, especially when persons are diagnosed in the late chronic stage of infection^51,52^. In the present analysis, DS was able to detect low-abundance viral variants with frequencies <10%, which were not detected by SS, however, most of the results were in complete agreement (n = 39/47, 82.9%). Partial concordance was observed in two samples where DS was able to detect all the mutations identified by SS and some additional SDRMs. Complete discordance was observed in six samples. In four samples discordance was due to the SDRM T215S being detected by DS at low frequencies and falling below threshold in SS. In the other two samples, highlighted by the disagreement between the platforms, the mutation patterns diverged completely. Similar results were reported by other studies, which showed that DS is able to identify SDRMs that are commonly not identified by SS and that minor disagreement between the two platforms is possible^53^. In this study, clinical significance of minority SDRMs was not evaluated, since we wanted to emphasize the importance of including new technologies in clinical diagnostics and combining them with already established ones, especially in complex clinical cases that do not respond to first-line treatment options.

In conclusion, this is the first comprehensive study on molecular epidemiology of HIV-1 infection in Croatia. We have shown that the epidemic is mainly local and driven by the young MSM population. We determined a high overall prevalence of SDRM of 16.4% and forward spread of resistant viral strains, even that of triple-class resistance. Most of the identified mutations were clinically relevant, indicating a need for baseline HIV resistance testing before treatment initiation in Croatia. Even though a low prevalence of resistance to InSTI was observed, TDR to InSTI needs to be carefully surveyed on a national level. In addition, we demonstrated that deep sequencing is a reliable tool for accurate identification of low-level viral quasispecies. These data can help develop prevention strategies and public health interventions. We suggest that in settings similar to that of Croatia, where high prevalence of SDRM is present and where most of the cohort is connected through transmission networks, new approaches, such as identification of transmission clusters “in real-time” and immediate intervention, could pose a method to prevent further expansion of HIV-1 epidemics.

## Methods

### Study population and samples

This study included treatment naive HIV-infected persons ≥18 years old who entered HIV clinical care at University Hospital for Infectious Diseases (UHID) in the time period between January 2014 and December 2017 and had plasma viraemia >1,000 HIV-1 RNA copies per ml. A total of 436 HIV-infected persons enrolled in clinical care in the studied period, off which 403 persons have met inclusion criteria and were included in the study, providing coverage of 92.4%. Recent infection was defined as having at least one of the following finding: negative HIV-1 test within 6 months, evolution of antigen and antibody tests (positive p24 antigen and negative or undetermined Western blot with subsequent confirmation of HIV diagnosis by HIV-1 RNA and/or positive Western blot), and symptoms of acute HIV-infection. Selected demographic and epidemiological data were obtained from the patient’s records. The study was approved by the Ethics Committee of UHID on July 16th 2015 and the study was performed in accordance with the Declaration of Helsinki. Informed consents were signed by all persons included in this study.

### HIV genotypic drug resistance testing by Sanger sequencing

HIV-1 RNA was extracted from 500 μl of patient’s plasma sample using the QIAmp Viral RNA Mini Kit (Qiagen, Hilden, Germany). Genotyping of the partial HIV *pol* gene was performed in two separate reactions: (1) sequencing of the HIV-1 protease and reverse transcriptase region; (2) sequencing of the HIV-1 integrase region.

For 403 persons the entire HIV-1 protease region (codons 1–99) and part of the reverse transcriptase region (codons 1–240) were amplified with one-step reverse transcriptase polymerase chain reaction (RT-PCR) by using SuperScript III One-Step RT-PCR System with Platinum *Taq* (Invitrogen, Carlsbad, CA) and the region-specific primer set^54^. Nested-PCR assay was carried out for samples that were negative with first round PCR by using HotStarTaq DNA Polymerase (Qiagen) and the inner primer set^54^. Obtained amplicons of 1017 bp were sequenced with BigDye Terminator v3.1 Cycle Sequencing Kit (Thermo Fisher Scientific, Waltham, MA) with a set of five primers to obtain bidirectional sequences^53^. Sequences were aligned and compared with the reference strain HIV-1 HXB2 (GenBank number K03455) by using Vector NTI software (Thermo Fisher Scientific). Primary resistance to antiretroviral drugs was defined as the presence of ≥1 mutation of the WHO SDRM list^35^. Clinically relevant resistance to NRTIs, NNRTIs or PIs was evaluated with Stanford University HIV Drug Resistance Database, Genotypic Resistance Interpretation Algorithm version 8.8^31^ and IAS Drug Resistance Mutation list^32^.

Analysis of resistance to InSTIs was performed for persons who entered clinical care at UHID during 2017. A total of 110 patients entered clinical care during 2017, of which 100 patients met the inclusion criteria as reported above and were included in this part of the study. The entire HIV-1 integrase region (codons 1–288) was amplified by using SuperScript IV One-Step RT-PCR System with Platinum *Taq* (Invitrogen) and the specific primer set (Supplementary Table S3). Amplicons of 864 bp were sequenced with BigDye Terminator V3.1 Cycle Sequencing Kit (Thermo Fisher Scientific) and a set of four primers to obtain bidirectional sequences (Supplementary Table S3). Sequences were aligned and compared with the reference strain HIV-1 HXB2 (GenBank number K03455) by using Vector NTI software (Thermo Fisher Scientific). Primary resistance to InSTIs was predicted with Stanford University HIV Drug Resistance Database, Genotypic Resistance Interpretation Algorithm version 8.8^31^.

HIV-1 subtypes were determined by several algorithms: Rega HIV-1 Subtyping Tool, version 3.0., jumping profile Hidden Markov Model (jpHMM), COntext-based Modelling for Expeditious Typing (COMET) and finally confirmed with phylogenetic analysis^55–57^.

### Deep sequencing analysis

To characterize HIV-1 minority drug resistance variants present at frequencies below the detection limit of Sanger sequencing, 48 persons were randomly selected for deep sequencing analysis. Part of the HIV *pol* gene that spans the whole HIV-1 protease region and part of the reverse transcriptase region (K03455 number for the gene specific position 2189–3753) and the region that spans the whole integrase gene (K03455 number for the gene specific position 4180–5200) were sequenced with MiniSeq (Illumina, San Diego, CA). HIV-1 RNA was extracted as reported above and reverse transcribed with SuperScript III First-Strand Synthesis System for RT-PCR (Invitrogen) and UNINEF primer^58^. Amplification of the target region for each sample was done in 4 separate multiplex PCR reactions using ALLin Taq DNA Polymerase (highQu, Kraichtal, Germany). For this purpose, we constructed 22 primer pairs that span the specific region of interest (Supplementary Table S4). After multiplex PCR, amplicons of each sample were pooled in one tube and purified with Agencourt Ampure XP beads (Beckman Coulter, Krefeld, Germany). Viral DNA libraries were prepared for deep sequencing with NEBNext Ultra II DNA Library Prep Kit for Illumina (New England BioLabs, Beverly, MA), according to the manufacturer’s instructions. Library concentrations and purity were measured with Agilent High Sensitivity Kit (Agilent Technologies, Santa Clara, CA) on Bioanalyzer 2100. Sequencing was performed using MiniSeq MID output 300 cycles reagent kit (paired-end; 150 + 150). Demultiplexed sequencing files were converted to FASTQ files and further analysed with HyDRA Web (Government of Canada, Ottawa, Canada)^59^. Data were analysed with 5% sensitivity threshold, default target coverage (10,000 reads) and default filtering settings (length cut-off: 100; score cut-off: 30; Minimum Variant Quality: 30; Minimum Read Depth: 100) incorporated in HyDRA^59^.

### Phylogenetic and phylodynamic analysis of Sanger sequences

To observe transmission networks, separate phylogenetic analyses were performed for most prevalent subtypes determined in this study. Namely, 368 sequences subtyped B, 17 sequences subtyped A and 7 sequences subtyped C were included in this part of the study. Ten most similar control sequences were included per each local sequence by searching the BLAST database^60^. After removing duplicates, 667 subtype B, 104 subtype A and 62 subtype C control sequences were included. In addition, three reference sequences of subtype A were included to root the subtype B and C phylogenetic trees and four reference sequences of subtype B to root subtype A phylogenetic tree. All sequences were aligned using CLUSTALW sequence alignment tool available in Bioedit, manually edited and trimmed to the size of 992 bp^61^. Phylogenetic inference was performed with PhyML 3.0 program with an integrated model selection following the Akaike Information Criterion (AIC)^62^. TC was defined as a group of three or more persons/sequences on the same branch of the phylogenetic tree with aLRT >0.90^63^. Local TCs were defined as clusters consisting of >75% of Croatian sequences, while mixed TCs were defined as clusters consisting of <75% of Croatian sequences. TCs within the phylogenetic trees were manually examined to ensure sequences from clusters grouped together with high branch support with using Figtree v1.4.3^64^. To rule out clustering bias due to the presence of drug resistance mutations (DRM), additional phylogenetic trees were constructed for each subtype: with the exclusion of DRM sites. Namely, 43 codons related to the major drug resistance mutations, according to the IAS list^32^, were removed: PR: 23, 24, 30, 32, 46, 47, 48, 50, 53, 54, 73, 76, 82, 83, 84, 85, 88, 90; RT: 41, 65, 67, 69, 70, 74, 75, 77, 100, 101, 103, 106, 115, 116, 151, 179, 181, 184, 188, 190, 210, 215, 219, 225, 230; resulting in the final sequence size of 863 bp.

To estimate the age of the most recent common ancestor (*t*MRCA, years) a Bayesian Markov Chain Monte Carlo (MCMC) approach was used and TCs with posterior probability 1 were further analysed. Since phylogenetic inference showed high rate of clustering among local sequences, we conducted separate analysis for 10 TCs with sample size varying from 9 to 61 sequences. Analysis of the whole dataset and selected TCs was done in order to compare their tMRCA values and to estimate population growth rate of each TC. We included only sequences without indication of recombination according to RDP3 analysis^65^. Out of a total of 363 subtype B sequences, four sequences were determined as potential recombinants and for eight sequences the exact sampling dates were unknown and were therefore removed from the analysis. The final dataset consisted of 354 local sequences and 571 time-stamped control sequences. Sampling date for all local sequences was defined as the date when the patient’s blood was drawn, while for the most background sequences the exact date was unknown and was defined as a midpoint of the sampling year, as reported in the Los Alamos HIV sequence database. The best fitting nucleotide substitution model was determined to be HKY + I + G in MEGA6 programme^66^. Phylodynamic analysis was performed with the BEAST2 package, v.2.1.3^67^, using the coalescent Bayesian skyline population model with a relaxed log-normal clock model^68^ and the HKY substitution model with 4 discrete gamma rates and invariant sites^69^. The MCMC was run in duplicate MCMC chains of 500,000,000 iterations each, with different initialization seed values and a sampling rate of 0.001. The first 10% of states sampled from each MCMC chain were discarded as burn-in and the duplicate chains were concatenated using LogCombiner^67^. The concatenated BEAST2 trace log-file was viewed in Tracer v.1.7.1^70^ and checked for effective sample size (ESS) values of > 200. The concatenated tree log-file was downsampled to 50,000 trees using LogCombiner and Maximum Clade Credibility Tree was prepared using TreeAnnotator^67^ and further visualised using FigTree v1.4.3^64^.

### Statistical analysis

Values were described by frequencies and percentages or median with first and third quartiles (Q1, Q3). Categorical variables between the two groups were compared using the odds ratio (OR) and 95% confidence intervals (CI) and by the chi-square or Fisher’s exact test, as appropriate. Continuous variables between the two groups were compared using Mann–Whitney *U* test. All statistical analyses were performed with the statistical software SAS version 9.4 (SAS Institute, Cary, NC, USA).

## Supplementary information

Supplementary information

## Data Availability

All data analysed in this study is included in this published article and its Supplementary Information file. All generated sequences by SS are available from the GeneBank database under accession numbers MN163320-MN163821, MN187252, while sequences generated by DS are available from ArrayExpress database at EMBL-EBI under accession number E-MTAB-8153.
