# Supplementary material for: Analysis of HIV-1 diversity, primary drug resistance and transmission networks in Croatia
Source: Sci Rep. 2019 Nov 21;9:17307. doi: 10.1038/s41598-019-53520-8 (PMC6872562; doi:10.1038/s41598-019-53520-8)
Supplement: Supplementary file 1 — Supplementary information [file 41598_2019_53520_MOESM1_ESM.doc]

**Analysis of HIV-1 diversity, primary drug resistance and transmission networks in Croatia**

Maja Oroz^1^, Josip Begovac^1,2^, Ana Planinić^2^, Filip Rokić^3^, Maja M. Lunar^5^, Tomaž Mark Zorec^5^, Robert Beluzić^3^, Petra Korać^4^, Oliver Vugrek^3^, Mario Poljak^5^, Snježana Židovec Lepej^2,4*^

1 University of Zagreb, School of Medicine, Zagreb, 10000, Croatia.

2 University Hospital for Infectious Diseases "Dr. Fran Mihaljević", Zagreb, 10000, Croatia.

3 Ruđer Bošković Institute, Zagreb, 10000, Croatia.

4 Division of Molecular Biology, Department of Biology, Faculty of Science, University of Zagreb, 10000, Croatia.

5 Institute of Microbiology and Immunology, Faculty of Medicine, University of Ljubljana, Ljubljana, 1000, Slovenia.

***** Correspondence to Snježana Židovec Lepej: szidovec@gmail.com

Table S1. Patterns of SDRMs in 66 treatment-naive HIV-1 persons in Croatia (2014 – 2017).

| Risk Factor | Age | Subtype | SDRM to NRTI | SDRM to NNRTI | SDRM to PI | TDRM to InSTI | Clinical relevance according to IAS list | Clinical relevance according to Stanford HIVdb |
| --- | --- | --- | --- | --- | --- | --- | --- | --- |
| MSM | 26 | B | T215D | K103N, L100I | V32I, I47V | ND | Clinically resistant strain | Intermediate to high-level resistance |
| MSM | 35 | B | T215D | K103N, L100I | V32I, I47V | ND | Clinically resistant strain | Intermediate to high-level resistance |
| MSM | 23 | B | M184MIV, T215D | K103N, L100I | V32I, I47V | ND | Clinically resistant strain | Intermediate to high-level resistance |
| MSM | 23 | B | T215E | K103N, L100I | V32I, I47V | / | Clinically resistant strain | Intermediate to high-level resistance |
| MSM | 29 | B | T215D | K103N, L100I | V32I, I47V | ND | Clinically resistant strain | Intermediate to high-level resistance |
| MSM | 30 | B | T215D | K103N, L100I | V32I, I47V | ND | Clinically resistant strain | Intermediate to high-level resistance |
| MSM | 37 | B | T215E | K103N, L100I | V32I, I47V | ND | Clinically resistant strain | Intermediate to high-level resistance |
| MSM | 42 | B | T215E | K103N, L100I | V32I, I47V | ND | Clinically resistant strain | Intermediate to high-level resistance |
| MSM | 36 | B | M184MIV, L210W, T215S | K101E, Y181C, G190A, P225PH | I84V | ND | Clinically resistant strain | Intermediate to high-level resistance |
| MSM | 32 | B | M41L, T215L | / | / | / | Clinically resistant strain | Low-level to intermediate resistance |
| MSM | 40 | B | M41L, T215L | / | / | / | Clinically resistant strain | Low-level to intermediate resistance |
| MSM | 54 | B | M41L, T215L | / | / | / | Clinically resistant strain | Low-level to intermediate resistance |
| MSM | 20 | B | L210W, T215S | / | / | ND | Clinically resistant strain | Low-level to intermediate resistance |
| MSM | 21 | B | L210W, T215S | / | / | ND | Clinically resistant strain | Low-level to intermediate resistance |
| MSM | 21 | B | L210W, T215S | / | / | ND | Clinically resistant strain | Low-level to intermediate resistance |
| MSM | 24 | B | T215S, L210W | / | / | ND | Clinically resistant strain | Low-level to intermediate resistance |
| MSM | 24 | B | L210W, T215S | / | / | ND | Clinically resistant strain | Low-level to intermediate resistance |
| MSM | 29 | B | L210W, T215S | / | / | / | Clinically resistant strain | Low-level to intermediate resistance |
| MSM | 39 | B | L210W, T215S, | / | / | / | Clinically resistant strain | Low-level to intermediate resistance |
| MSM | 42 | B | L210W, T215S | / | / | / | Clinically resistant strain | Low-level to intermediate resistance |
| MSM | 47 | B | L210W, T215S | / | / | / | Clinically resistant strain | Low-level to intermediate resistance |
| MSM | 41 | B | T215S | / | / | ND | Susceptible | Low-level resistance |
| MSM | 33 | B | T215S | / | / | ND | Susceptible | Low-level resistance |
| MSM | 20 | B | T215S | / | / | ND | Susceptible | Low-level resistance |
| MSM | 38 | B | T215S | / | / | ND | Susceptible | Low-level resistance |
| MSM | 39 | B | T215S | / | / | ND | Susceptible | Low-level resistance |
| MSM | 22 | B | T215S | / | / | ND | Susceptible | Low-level resistance |
| MSM | 25 | B | T215S | / | / | ND | Susceptible | Low-level resistance |
| MSM | 26 | B | T215S | / | / | ND | Susceptible | Low-level resistance |
| MSM | 26 | B | T215S | / | / | ND | Susceptible | Low-level resistance |
| MSM | 32 | B | T215S | / | / | ND | Susceptible | Low-level resistance |
| MSM | 33 | B | T215S | / | / | ND | Susceptible | Low-level resistance |
| MSM | 35 | B | T215S | / | / | ND | Susceptible | Low-level resistance |
| MSM | 37 | B | T215S | / | / | ND | Susceptible | Low-level resistance |
| MSM | 37 | B | T215S | / | / | ND | Susceptible | Low-level resistance |
| MSM | 37 | B | T215S | / | / | ND | Susceptible | Low-level resistance |
| MSM | 43 | B | T215S | / | / | ND | Susceptible | Low-level resistance |
| MSM | 47 | B | T215S | / | / | ND | Susceptible | Low-level resistance |
| MSM | 53 | B | T215S | / | / | ND | Susceptible | Low-level resistance |
| MSM | 62 | B | T215S | / | / | ND | Susceptible | Low-level resistance |
| MSM | 56 | B | T215S | / | / | ND | Susceptible | Low-level resistance |
| MSM | 21 | B | / | K101E | / | ND | Clinically resistant strain | Low-level to intermediate resistance |
| MSM | 30 | B | / | K101E | / | ND | Clinically resistant strain | Low-level to intermediate resistance |
| MSM | 44 | B | / | K101E | / | ND | Clinically resistant strain | Low-level to intermediate resistance |
| MSM | 37 | B | / | K101E | / | ND | Clinically resistant strain | Low-level to intermediate resistance |
| MSM | 40 | B | / | K101E | / | ND | Clinically resistant strain | Low-level to intermediate resistance |
| MSM | 46 | B | / | K101E | / | ND | Clinically resistant strain | Low-level to intermediate resistance |
| MSM | 48 | B | / | K101E | / | ND | Clinically resistant strain | Low-level to intermediate resistance |
| MSM | 48 | B | / | K101E | / | ND | Clinically resistant strain | Low-level to intermediate resistance |
| MSM | 54 | B | / | K101E | / | / | Clinically resistant strain | Low-level to intermediate resistance |
| MSM | 54 | B | / | K101E | / | / | Clinically resistant strain | Low-level to intermediate resistance |
| MSM | 59 | B | / | K101E | / | / | Clinically resistant strain | Low-level to intermediate resistance |
| MSM | 71 | B | / | K101E | / | / | Clinically resistant strain | Low-level to intermediate resistance |
| MSM | 52 | B | / | K101E | / | / | Clinically resistant strain | Low-level to intermediate resistance |
| MSM | 47 | B | / | K101E | / | / | Clinically resistant strain | Low-level to intermediate resistance |
| MSM | 28 | B | / | K103N | / | ND | Clinically resistant strain | High-level resistance |
| MSM | 44 | C | / | K103N | / | ND | Clinically resistant strain | High-level resistance |
| HETERO | 28 | B | L210W, T215D | / | / | ND | Clinically resistant strain | Low-level to intermediate resistance |
| MSM | 45 | B | K219R, M41L | / | / | ND | Clinically resistant strain | Low-level resistance |
| MSM | 47 | B | M41L, T215D | / | / | ND | Clinically resistant strain | Low-level to intermediate resistance |
| MSM | 52 | B | K219Q | / | / | ND | Clinically resistant strain | Potential low-level resistance |
| MSM | 29 | B | / | G190A | / | ND | Clinically resistant strain | Low-level to high-level resistance |
| MSM | 25 | B | / | G190E | / | / | Susceptible | Intermediate to high-level resistance |
| MSM | 29 | B | T69D | / | / | ND | Susceptible | Susceptible |
| MSM | 41 | A1 | / | / | M46I | / | Clinically resistant strain | Potential low-level resistance |
| MSM | 26 | B | / |  | / | G140A | Susceptible | Low-level to intermediate resistance |

MSM: men who have sex with men; SDRM: surveillance drug resistance mutation; TDRM: transmitted drug resistance mutation; NRTI: nucleoside reverse transcriptase inhibitors; NNRTI: non-nucleoside reverse transcriptase inhibitors; PI: protease inhibitors; InSTI: integrase strand-transfer inhibitors; ND: not done.

Table S2. Comparison of SDRMs detected by Sanger sequencing and deep sequencing.

| Accession numbers DS/SS | Mutation concordance | SS  SDRM | DS  SDRM | Frequencyof SDRM by DS,  n (%) | DS Coverage, number of reads |
| --- | --- | --- | --- | --- | --- |
| E-MTAB 8153/MN163599 | complete | T215S | T215S | 63.1 | 20572 |
| E-MTAB-8153/MN163471 | complete | T215S | T215S | 76.7 | 34493 |
| E-MTAB-8153/MN163490 | complete | T215S | T215S | 16.7 | 2104 |
| E-MTAB-8153/MN163505 | complete | T215S | T215S | 41.8 | 28781 |
| E-MTAB-8153/MN163615 | complete | T215S  L210W | T215S  L210W | 21.6  36.6 | 1288  1288 |
| E-MTAB-8153/MN163486 | complete | T215S  L210W | T215S  L210W | 21.4  15.8 | 3320  3320 |
| E-MTAB-8153/MN163535 | complete | K101E | K101E | 18.5 | 36017 |
| E-MTAB-8153/MN163622 | complete | K101E | K101E | 92.7 | 42749 |
| E-MTAB-8153/MN163553 | partial | V32I,  I47V  L100I  K103N  T215E | V32I  I47V  L100I  K103N  T215E  N348I | 17.9  43.1  32.1  32.6  38.3  28.9 | 1082  288  685  682  5147  236 |
| E-MTAB-8153/MN163437 | partial | V32I,  I47V  L100I  K103N  T215E | V32I,  I47V  L100I  K103N  T215E  T215S  T215D  Y181C  Y181H  N348I | 29.9  87.6  84.5  83.6  67.9  5.2  10.2  10.8  10.8  84.7 | 2385  1658  3673  3684  20732  20732  20732  59276  59276  2578 |
| E-MTAB-8153/MN163561 | divergent | / | T215S | 11.6 | 3637 |
| E-MTAB-8153/MN163517 | divergent | / | T215S | 10.1 | 3207 |
| E-MTAB-8153/MN163504 | divergent | / | T215S | 9.5 | 21941 |
| E-MTAB-8153/MN163607 | divergent | / | T215S | 9.4 | 7722 |
| E-MTAB-8153/MN163766 | divergent | G140A | I47V  T215D  T215S | 7.7  16.99  7.1 | 2162  9709  9709 |
| E-MTAB-8153/MN163457 | divergent | K219R  M41L | T215S | 25.5 | 47783 |

SDRM: surveillance drug resistance mutation; DS: deep sequencing; SS: Sanger sequencing.

Table S3. Primers for PCR and Sanger sequencing of HIV-1 integrase region (codons 1-288).

| **Primers for PCR** | **Sequence (5'->3')** |
| --- | --- |
| INT-1 forward | GGACATATCAAATTTATCAAGAGCC |
| INT-2 reverse | TGAGGGCTTTCATAGTGATGTC |
| **Primers for SS** |  |
| INT-3 forward | GGACATATCAAATTTATCAAGAGCC |
| INT-4 reverse | CCTACAATCCCCAAAGTCAAG |
| INT-5 forward | CAGCAATTTCACCGGTGCTA |
| INT-6 reverse | TGAGGGCTTTCATAGTGATGTC |

SS: Sanger sequencing

Table S4. Primers for deep sequencing library of HIV-1 reverse transcriptase (codons 1-400), protease (codons 1-99) and integrase (codons 1-288) region.

| **Primers for HIV-1 PI and RT region** | **FW primer, sequence (5'->3')** | **RV primer, sequence (5'->3')** |
| --- | --- | --- |
| Primer 1 | AGCCGATAGACAAGGAACTG | GATAAAACCTCCAATTCCCCCTAT |
| Primer 2 | GCTCTATTAGATACAGGAGCAGAT | CAATTATGTTGACAGGTGTAGGT |
| Primer 3 | GAAATCTGTGGACATAAAGCTATAGG | GGCCATTGTTTAACTTTTGGG |
| Primer 4 | TAGCCCTATTGAGACTGTACCAG | ACTGGAGTATTGTATGGATTTTCAG |
| Primer 5 | GTACAGAGATGGAAAAGGAAGG | CGGGATGTGGTATTCCTAATTG |
| Primer 6 | GAGAACTCAAGACTTCTGGGAAG | GTACTGATATCTAATCCCTGGTGTC |
| Primer 7 | ACAGTACTGGATGTGGGTGATG | AGGCTCTAAGATTTTTGTCATGCTAC |
| Primer 8 | TGTGCTTCCACAGGGATG | AGATGTTGTCTCAGCTCCTC |
| Primer 9 | GGATCTGACTTAGAAATAGGGC | AGCACTATAGGCTGTACTGTC |
| Primer 10 | CATCAGAAAGAACCTCCATTCCTT | CTTTAATCCCTGGGTAAATCTGACT |
| Primer 11 | CAATGACATACAGAAGTTAGTGG | GGTCATAATACACTCCATGTACTG |
| Primer 12 | CACTAACAGAAGAAGCAGAGCTAG | CCCTCATTCTTGCATATTTTCCTGT |
| Primer 13 | GGACATATCAAATTTATCAAGAGCC | ACCATGTTTCCCATGTTTCC |
| **Primers for HIV-1 IN region** | **FW primer, sequence (5'->3')** | **RV primer, sequence (5'->3')** |
| Primer 14 | GAATTGGAGGAAATGAACAAGTAG | GCTGACATTTATCACAGCTGG |
| Primer 15 | GGCTAGTGATTTTAACCTGCC | CACTGGCTACATGAACTGCT |
| Primer 16 | GCATGGACAAGTAGACTGTAGT | GTTTTTAGGCCATCTTCCTGC |
| Primer 17 | GAAACAGGGCAGGAAACAGC | CTTGACTTTGGGGATTGTAGG |
| Primer 18 | CAGCAATTTCACCGGTGCT | GATGAATACTGCCATTTGTACTGC |
| Primer 19 | GGTAAGAGATCAGGCTGAACAT | GCTGTCCCTGTAATAAACCCG |
| Primer 20 | GGGGAAAGAATAGTAGACATAATAGCAAC | GTCACTATTATCTTGTATTACTACTGCCC |
| Primer 21 | AAGCTCCTCTGGAAAGGTGAAG | CCATGTTCTAATCCTCATCCTGT |
| Primer 22 | TGCCAAGAAGAAAAGCAAAGATC | TGAGGGCTTTCATAGTGATGT |

PI: protease; RT: reverse transcriptase; IN: integrase; FW: forward; RV: reverse.


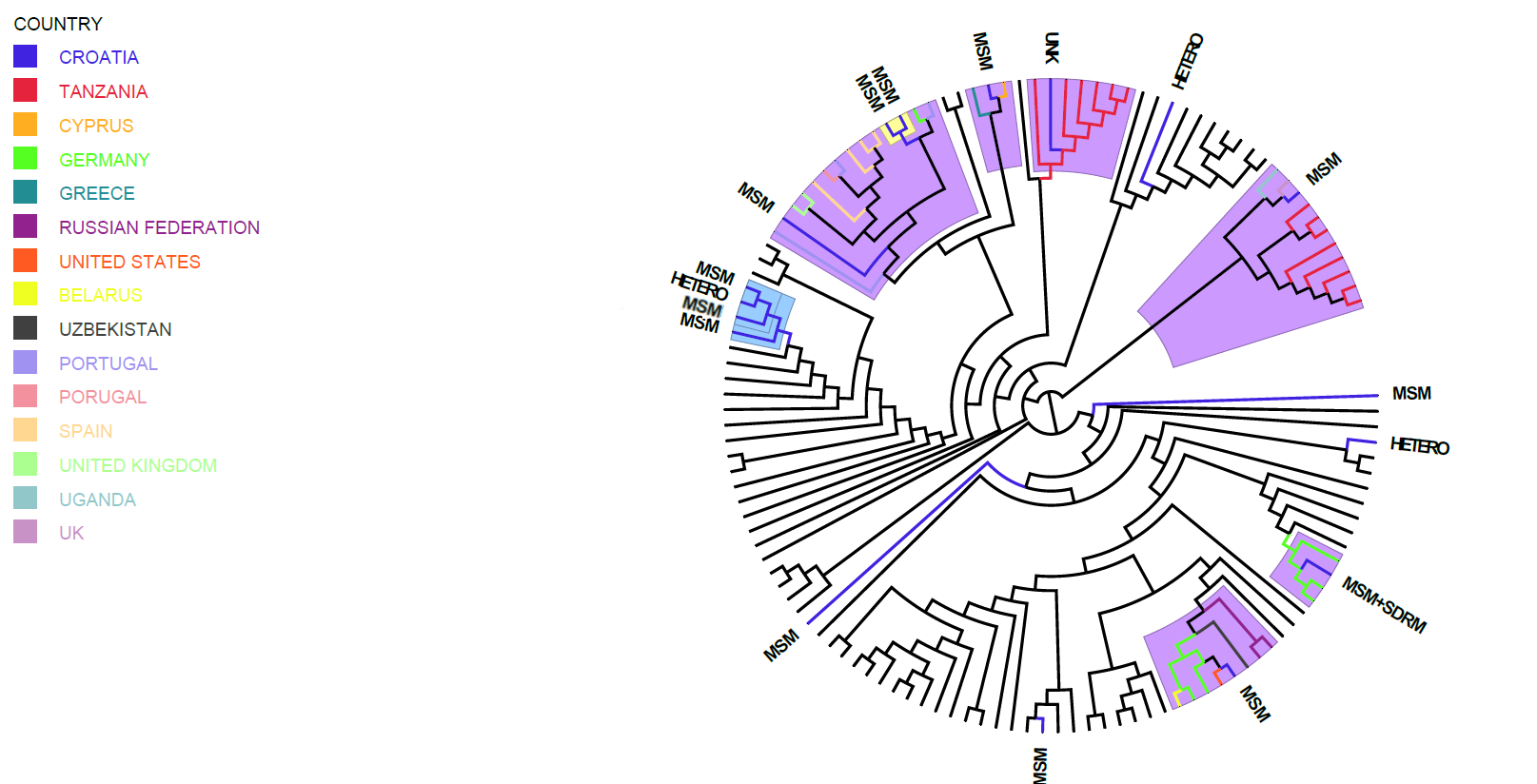
**Figure S1.** Maximum likelihood phylogenetic tree of the Croatian HIV-1 sequences subtype A1, corresponding background sequences and their geographic origin. Branches of Croatian and background sequences that participate in transmission clusters (TCs) are coloured according to the geographic origin, as presented on a legend at the Figure S1. All background sequences outside TCs are coloured black. For all Croatian sequences transmission risk is marked on the tree: MSM, men who have sex with men; HETERO, heterosexual; UNK, unknown. Sequences carrying surveillance drug resistance mutation (SDRM) are marked on the tree along with transmission risk. TCs with >75% of Croatian sequences (local clusters) are highlighted blue, while TCs with <75% of Croatian sequences (mixed clusters) are highlighted purple.


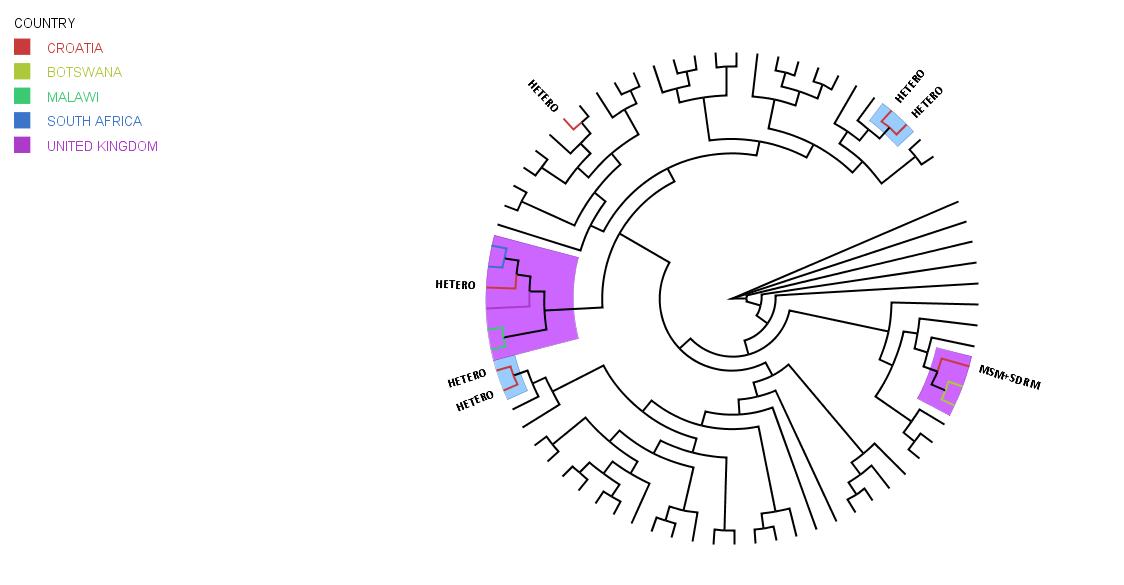
**Figure S2.** Maximum likelihood phylogenetic tree of the Croatian HIV-1 sequences subtype C, corresponding background sequences and their geographic origin. Branches of Croatian and background sequences that participate in transmission clusters (TCs) are coloured according to the geographic origin, as presented on a legend at the Figure S2. All background sequences outside TCs are coloured black. For all Croatian sequences transmission risk is marked on the tree: MSM, men who have sex with men; HETERO, heterosexual. Sequences carrying surveillance drug resistance mutation (SDRM) are marked on the tree along with transmission risk. TCs with >75% of Croatian sequences (local clusters) are highlighted blue, while TCs with <75% of Croatian sequences (mixed clusters) are highlighted purple.


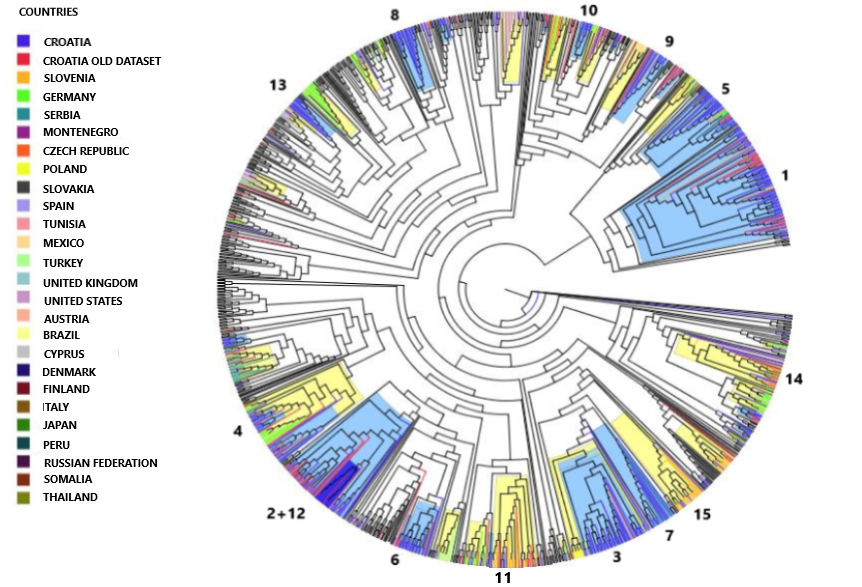
**Figure S3.** Maximum likelihood phylogenetic analysis of the Croatian HIV-1 sequences subtype B from the old (2006-2008), new (2014-2017) datasets and background sequences. Branches of Croatian sequences and all background sequences that participate in transmission clusters (TCs) are coloured according to the geographic origin, as presented on a legend at the Figure S3. All background sequences outside TCs are coloured black. TCs with >75% of Croatian sequences (local clusters) are highlighted blue, while TCs with <75% of Croatian sequences (mixed clusters) are highlighted yellow. TCs with ≥5 Croatian sequences (Table 3) are marked on the sideward by ordinal numbers.
